# Reuniting the Three Sisters: collaborative science with Native growers to improve soil and community health

**DOI:** 10.1007/s10460-022-10336-z

**Published:** 2022-07-17

**Authors:** D. G. Kapayou, E. M. Herrighty, C. Gish Hill, V. Cano Camacho, A. Nair, D. M. Winham, M. D. McDaniel

**Affiliations:** 1 World Languages and Cultures, 505 Morrill Road, 2232 Pearson Hall, Ames, IA 50011 USA; 2 Sustainable Agriculture, 2200 Osborn Drive, 137 Bessey Hall, Ames, IA 50011 USA; 3 Agronomy, 716 Farm House Lane, 2104 Agronomy Hall, Ames, IA 50011 USA; 4 Food Science and Human Nutrition, 2302 Osborn Drive, 220 MacKay Hall, Ames, IA 50011 USA

**Keywords:** 3 Sisters, Food sovereignty, Intercropping, Indigenous agriculture, Milpa, Native american, Niche complementarity, Rematriation, Seed sovereignty, Traditional ecological knowledge

## Abstract

Before Euro-American settlement, many Native American nations intercropped maize (*Zea mays*), beans (*Phaseolus vulgaris*), and squash (*Cucurbita pepo*) in what is colloquially called the “Three Sisters.” Here we review the historic importance and consequences of rejuvenation of Three Sisters intercropping (3SI), outline a framework to engage Native growers in community science with positive feedbacks to university research, and present preliminary findings from ethnography and a randomized, replicated 3SI experiment. We developed mutually beneficial collaborative research agendas with four Midwestern US Native American nations. Ethnographic data highlighted a culturally based respect for 3SI as living beings, the importance it holds for all cultural facets of these Native nations, and the critical impact the practice has on environmental sustainability. One concern expressed by Native growers during ethnographic research was improving soil health—part of the rationale for establishing the 3SI agronomic experiment. To address this, we collaboratively designed a 3SI experiment. After 1 year, 3SI increased short-term soil respiration by 24%, decreased salt-extractable nitrate by 54%, had no effect on soil microbial biomass (but increased its carbon-to-nitrogen ratio by 32%) compared to the average of monoculture crops. The overarching purpose of this collaborative project is to develop a deeper understanding of 3SI, its cultural importance to Native communities, and how reinvigorating the practice—and intercropping in general—can make agroecosystems more sustainable for people and the environment.

## Introduction

Agriculture has evolved and taken many forms since first arising in human history at ~ 12,000–23,000 years ago (Snir et al. 2015). More recently, a focus on productivity has created high-yielding agroecosystems with 1 to 1.5% increase in grain yield per year for the world’s four major grain crops (Tilman et al. 2011; Ray et al. 2013). However, this rise in agricultural productivity with conventional industrialized agriculture has come with consequences. Some of these issues include: impaired water quality (Rabalais et al. 2001; Broussard and Turner 2009), rampant soil erosion (Montgomery 2007; Gelder et al. 2018), losses of soil organic matter (SOM)—the cornerstone of most soil ecosystem services—by 18–60% (Guo and Gifford 2002; De et al. 2020), increased grower reliance on agriculture inputs (Ward 1993; Tilman et al. 2002; Zhang 2018), and the negative consequences of climate change (Ray et al. 2019). Ironically, these unintended environmental consequences driven by myopic focus on productivity may actually have hindered agriculture’s ability to reduce global hunger.

The plant ecologist Robin Kimmerer (Citizen Potawatomi) has encouraged growers to rethink agroecosystem management to reflect the “honorable harvest” a covenant of reciprocity between humans and the land (Kimmerer 2013, 2017). This covenant entails recognizing the connection between humans and the natural world (i.e., ecology), emphasizing gratitude for nature’s gifts, and need for a reciprocal relationship with nature whereby we cannot take without giving. The honorable harvest has been practiced thousands of years in Native America and other parts of the globe (Dublin and Tanaka 2014; Lincoln 2019), but the practice has deteriorated through industrialization of agriculture within the last few centuries. The importance of reciprocity and the honorable harvest are also reflected in related ideas that are now part of a broader agroecological movement, including soil health (Karlen 2020; Karlen et al. 2021), ecological nutrient management (Drinkwater 2009; Drinkwater et al. 2017), and regenerative agriculture (Pearson 2007).

Scholars are increasingly turning to Traditional Ecological Knowledge (TEK) as a vital source of knowledge that can inform Westernized science and its applications (Berkes et al. 1994, 2000; Berkes and Usher 2000). The historical agricultural practice of planting crops in close proximity to each other—otherwise known as intercropping—leverages ecological principles to improve current agroecosystem services. While central to the TEK of some Native nations, intercropping has diminished over the decades.

Intercropping enhances agroecosystem services by filling ecological niches and increasing plant diversity compared to monoculture cropping. Thus, the historical practice of intercropping can also inform modern ecological nutrient management. For example, many Native American agriculturalists intercropped maize (*Zea mays*), beans (*Phaseolus vulgaris*), squash (*Cucurbita pepo*), and sometimes sunflowers (*Helianthus annuus*)—called the three (or four) sisters—because these crops were observed to thrive together (Mt. Pleasant 2006; Landon 2008; Kimmerer 2013; LaDuke 2019). Contemporary findings have demonstrated that the Three Sisters also increase human dietary diversity (Ruel 2003; Luna-González and Sørensen 2018; Lopez-Ridaura et al. 2021). The combination of beans, maize and squash provides complementary proteins, vitamins and minerals for human nutrition (USDA-ARS 2019).

### Brief agricultural history of Native America

Native North Americans were cultivating crops for generations before European contact, with some estimates as early as ~ 5000 BCE (Hurt 1987; Green and Arzigian 1994; Landon 2008). Native nations in the northeast, southeast, southwest, the Great Lakes, and the Plains historically grew multiple crops, including corn, beans, squash, sunflowers, and tobacco. Ecologist Kat Anderson has demonstrated that Native people in regions without agriculture, such as California and the Great Basin, were nonetheless “tending the wild” in a manner that is agricultural in nature (Anderson 2005). Prior to colonization, TEK laid the foundation for Native American growers to recognize environmental and management factors that resulted in optimal agroecological outcomes. Archeologists suggest 1000 BCE for the earliest beginnings of pre-maize agriculture in the Midwest US (Fritz 1990, 1995; Emerson et al. 2020). Before the introduction of maize, Native nations in the region were growing crops like Goosefoot (*Chenopodium* sp.), squash, gourds (*Cucurbita* and *Lagenaria* spp.), and sunflowers (*Helianthus annuus*) (Smith et al. 2007). These crops would have been planted within woodland clearings (Gartner 1999), on floodplain soils along rivers and streams due to inherent fertility (Gallagher et al. 1985; Sasso 2003), and sometimes even within wetlands (Doolittle 1992). Garden sizes ranged from a small plot meant to feed a family to large fields spanning hundreds of hectares (Doolittle 1992).

Spreading from its origins in Mesoamerica, growing the Three Sisters became increasingly common in the Midwest and Northeast by the middle 1500s (Mt. Pleasant 2006; Landon 2008; Cicarelli 2012). The TEK of growing maize with her two or Three Sisters was developed from thousands of years of observation and experimentation, passing the agroecological information gathered down from one generation to the next (Snively and Corsiglia 2001). Native growers observed that intercropping maize, beans, and squash proved advantageous to monocropped fields (Folk 1995; Mt. Pleasant 2016). Growing multiple species also bolstered agricultural resilience against weather extremes (Miewald 1995). Furthermore, evidence of territorial social responsibility may explain wide adoption (and success) of the intercropping practice because of its social and environmental sustainability (Morrow et al. 2018; Rusciano et al. 2019).

### Challenges for Native American agriculturalists resulting from colonization

When Europeans first contacted Native peoples, in South American, Mesoamerica, and North America, they were astounded by the productivity of the cropping systems in each location. Two hundred years ago, crop production by Native American agriculturalists around the Great Lakes region and along the Missouri and Red Rivers sustained the US fur trade and therefore contributed to broader global economies (Wessel 1976; Jablow 1994; Geniusz 2015). Even though they placed a high value on Native American agricultural trade, eventually Euro-Americans settled permanently on the most fertile land, usurped carefully developed seeds, and imposed policies and agricultural practices that made Native agriculture nearly impossible (Hurt 1987; Kipp 1988; Gewertz and Errington 2017).

The policy of removal was a major factor undermining Native agricultural practices. Forcing Native peoples from their homelands (with often more fertile conditions that they were adapted to) onto marginal lands had detrimental effects on Native agriculture (Hurt 1987; Kipp 1988). The policies establishing reservations assigned Euro-American farmers to pressure Native men to practice new forms of agriculture (Hurt 1987; Kipp 1988; Carlson 1992). The 1887 Allotment Act assigned small plots to nuclear families, which was not the basic unit of Native community structure, further limiting access to lands and preventing community farming practices (Carlson 1981; Hurt 1987). Assimilationist education policy sent Native children to boarding schools, where they had no opportunity to learn Native agriculture techniques or preservation and preparation of Native foods (Littlefield 1996; Bess 2013). By the 1930s, Three Sisters agriculture had been almost entirely eradicated from Native communities in the US Midwest. Much of the agricultural TEK centered in the Midwest has also been erased from the dominant national historical narrative, which focused on the Northeast and the Southwest centers of Native agricultural production.

### Modern agronomic benefits of intercropping

Mounting evidence has demonstrated the many agronomic benefits of intercropping, despite its increasingly infrequent use. Intercropping with polycultures has advantages compared to monocrop or crops diversified through time via rotations. First, intercropping with a variety of plant resource acquisition strategies (i.e., diversity in root and shoot architectures) may promote more efficient use of resources compared to monocrop in what is called niche complementarity (MacArthur and Levins 1967; Loreau and Hector 2001). Second, diversifying plant phenotypes through intercropping may have positive belowground effects on soil biota that create positive plant-soil feedbacks (Eisenhauer 2012). A few recent meta-analyses show that intercropping provides a 22 to 32% yield advantage compared to monocrops on average, when yield is normalized for required land area (Yu et al. 2015; Martin-Guay et al. 2018; Xu et al. 2020). There is also some evidence for belowground benefits of intercropping as well (Zhang et al. 2014; Wang et al. 2015), though the mechanisms for these positive effects remain largely unknown.

### Potential for Three Sisters intercropping to revitalize and regenerate

**Fig. 1:**
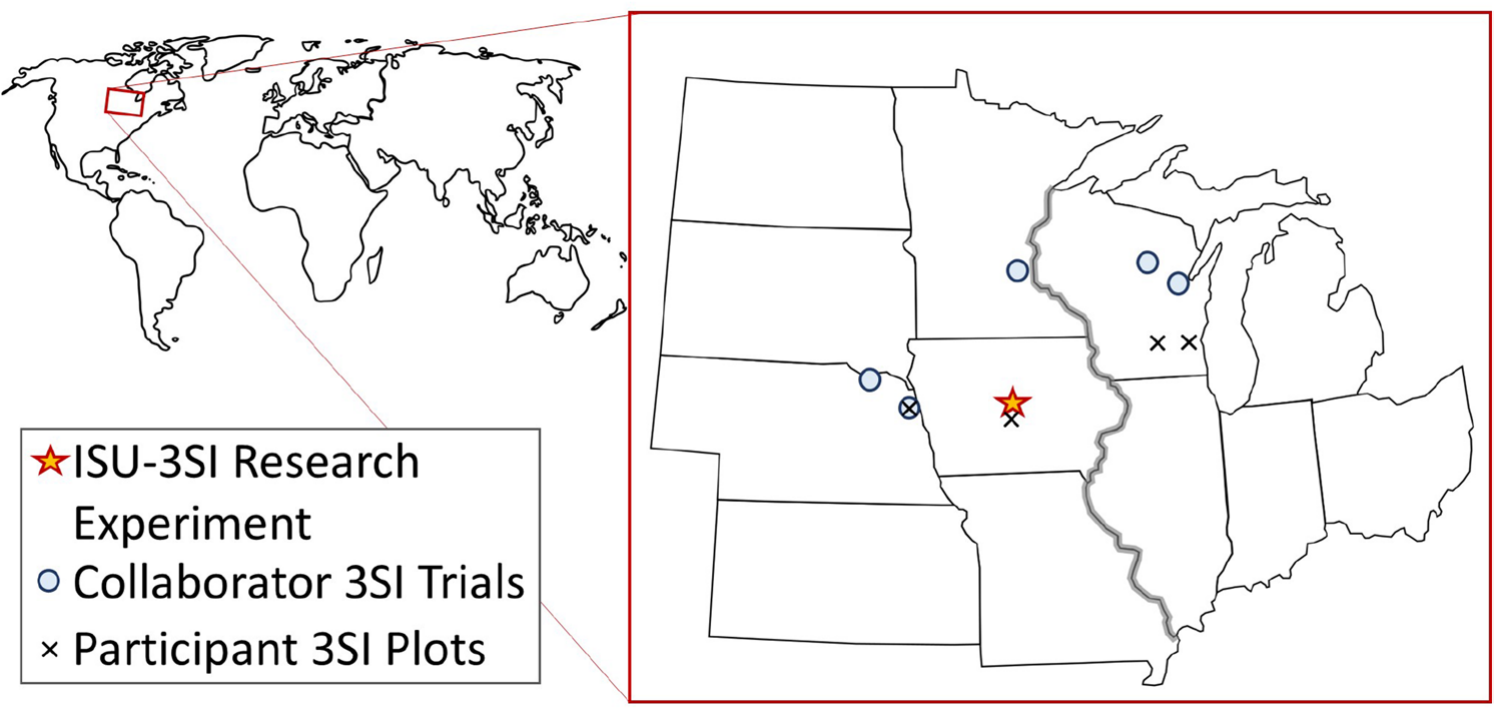
Map of the Midwest US showing locations of the Iowa State University Three Sisters intercropping experiment (ISU-3SI), Collaborator 3SI trials, and Participants growing Three Sisters in backyard gardens. The main research experiment is located at Iowa State University Horticulture Research Station near Story City, IA

**Fig. 2:**
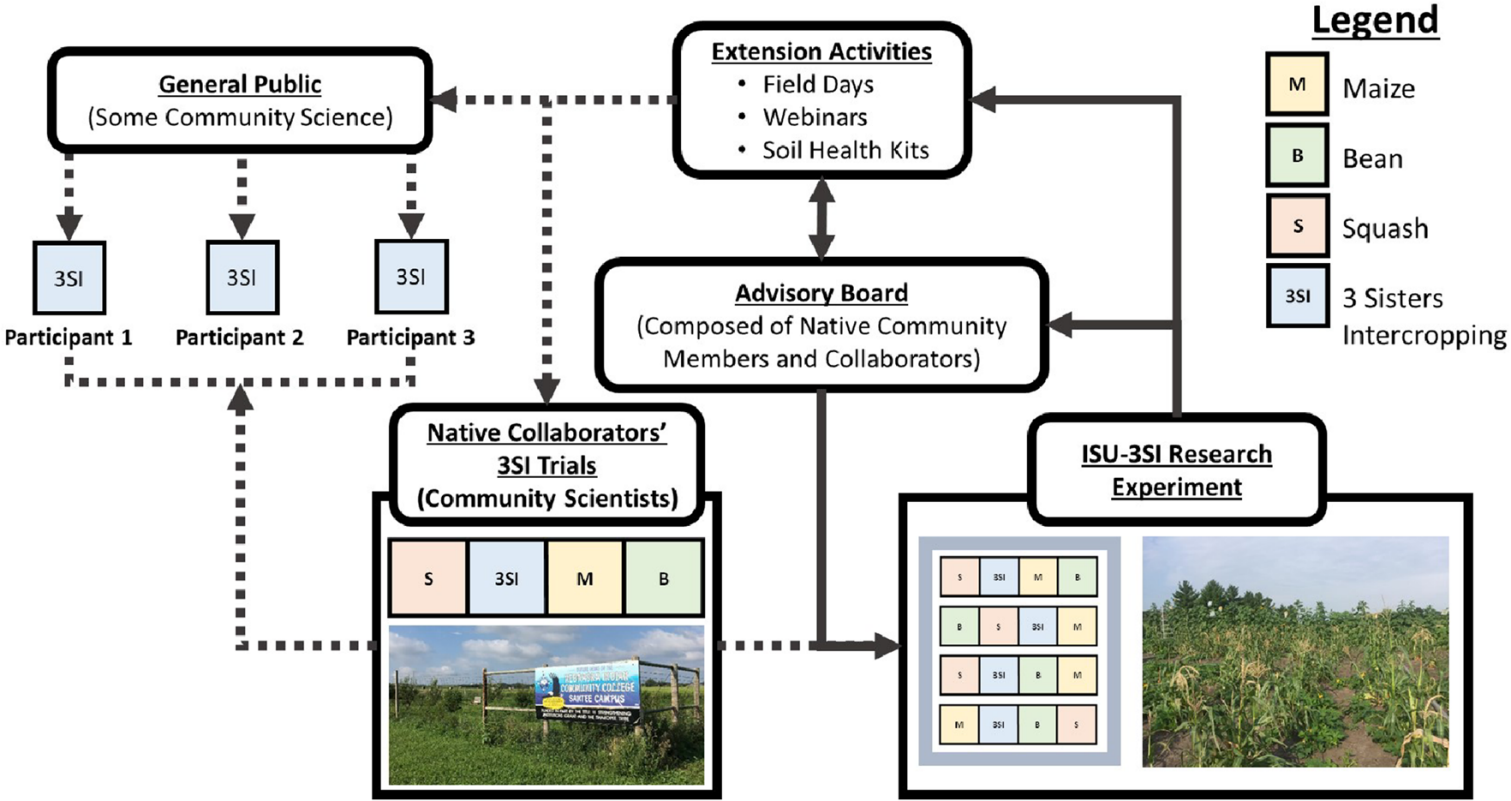
Conceptual diagram showing the importance of collaborative science and public outreach in the Three Sisters Intercropping Network (3SI-Net) project. The Iowa State University 3SI Research Experiment (ISU-3SI) receives input from an Advisory Board composed of Native Americans (many of whom are also Collaborators or Participants). This feedback between the ISU-3SI and Advisory Board drives the logistics and research agenda at the main research experiment and extension activities that engage Native Collaborators and Participants wanting to grow the Three Sisters and/or conduct DIY soil health measurements. Photos: Left, M.D. McDaniel photo of Nebraska Indian Community College garden in Santee, NE; Right, E.M. Herrighty photo of ISU-3SI Research Experiment near Story City, IA

Given the TEK on Three Sisters Intercropping (3SI) and modern evidence supporting diversified intercropping, we sought to explore both the cultural and agronomic underpinnings of 3SI in collaboration with Native growers in several Midwestern US states (Fig. 1). Currently we are collaborating with Native growers from the Nebraska Indian Community College, serving the Santee Sioux reservation, the Omaha reservation, and the Sioux City urban Native community, Dream of Wild Health, serving the Twin Cities Native community, the Oneida nation of Wisconsin, and the Menominee nation of Wisconsin. Our project—the Three Sisters Intercropping Network (3SI-Net)—uses an inclusive research approach with an Advisory Board, Native Collaborators, and growing list of Participants (many of which live on reservations or in urban Native communities) (Fig. 2). Our long-term goals with the project are to:

1. improve nutrition, environmental sustainability, and soil health by supporting Native American efforts to reinvigorate the practice of 3SI.
2. rematriate seeds that are no longer easily available to Native Communities.
3. understand the biophysical benefits of the 3SI practice to improve sustainability of conventional, industrialized agriculture which currently relies heavily on monoculture cropping systems.

For this paper, we introduce the larger 3SI-Net project and report preliminary results. First, we discuss ethnographic methods describing the importance of 3SI and soils to Native American communities, some current obstacles to revitalization, and the potential for revitalizing 3SI to improve nutrition and rural community economics. This ethnographic data helped us in collaboration with our Advisory Board to develop our 3SI agronomic experiment. Second, we present the first-year effects of 3SI on crop productivity, soil nutrients, microbial biomass, and microbial activity from a randomized complete block design experiment. We hypothesized that 3SI will: (i) have comparable or greater yields when compared to percentage of total monoculture when expressed on a land equivalent ratio, (ii) have enhanced nutritional value in edible portion of crops due to more efficient nutrient uptake, (iii) increase soil microbial biomass and activity compared to monoculture crops, (iv) decrease plant-available nutrients in the soil due to a more effective plant uptake of nutrients due to enhanced foraging from diversity of roots facilitating niche partitioning compared to monoculture crops.

#### Materials and methods

### Collaborative research approach with Indigenous communities

Building relationships based on trust is essential to conducting ethnography and establishing collaborative research with Native growers, requiring us to utilize Indigenous methodologies. Over the last two decades, Indigenous scholars have developed methods for conducting research within their communities (Corntassel et al. 2009; Kovach 2010; Smith 2013) Using methodologies developed in collaboration with Native communities, we work to ensure that our research serves the priorities of Native Nations (Ninomiya et al. 2020; Botha 2011; Burnette et al. 2014). To build a collaborative research project, Native people must participate in designing the research and they must be invested in the outcome of the research (Kovach 2010; Smith 2013). This demands respect for Native people as experts in their own right and purveyors of TEK. The benefit of the research to the Native community must also be clear (Smith 2013). This project provides soil testing, extension workshops on topics of interest to Native growers, and rematriation of rare Indigenous seeds to the communities from which they can be traced as mechanisms to ensure that this research benefits Native growers and wider Native food sovereignty efforts.

To confirm that Native perspectives shape our research and extension, we created an advisory board comprised of Native growers that meets at least once a year to provide direct input (Fig. 2). We also consult with members throughout the year as new questions arise. Drawing on the Principles for Indigenous Data Governance, the Advisory Board helps to design the experimental research conducted at Iowa State University (ISU), select the Indigenous seeds grown in the 3SI plots, and ensure that the resulting data and plant materials are treated in a culturally appropriate manner (Carroll et al. 2019). Initially Gish Hill reached out to the Indigenous Seed Keepers network to share an index of Native affiliated seeds held at the USDA Plant Introduction Station in Ames, Iowa, and available through the Germplasm Resource Information Network (GRIN) to share resources with Native growers with no expectation of participation. This initial sharing of information opened the door to wider discussions of the potential for collaborative research. As part of this relationship building, we visited growers, spent time on their farms listening to their concerns, and offered our services (such as soil testing and help with harvest).

#### The importance of seeds and selection for rematriation

Throughout the early planning stages of this project, we became aware of a crucial need within Native communities for access to their culturally significant seeds (Herrighty 2022). This rising food/seed sovereignty movement within Indigenous efforts to reclaim ancestral seeds is termed “seed rematriation.” Many cherished varieties with known ties to particular tribal nations are currently stored in the public and private seedbanks of non-Native institutions (White 2018). Thus, seed rematriation became an integral aspect of the 3SI-Net project when we realized our privilege and capacity to lend to rematriation efforts. The USDA National Plant Germplasm System is one of the institutions that stewards ancestral Native varieties. This service sends seed samples to researchers, primarily plant breeders, at no cost (USDA-ARS 2020). Since our agronomic research on 3SI requires Native seed varieties, we were able to leverage the seed acquisitions and reproduce them to be rematriated as part of the Iowa State University 3SI Research Experiment (ISU-3SI) described later (Table 1).

**Table 1:** Information on crops included in the Three Sisters intercropping experiment (and sunflower border)

Scientific name	Cultivar/variety name	Tribal affiliation	Selection criteria	Seed source	Number planted per mound
*Zea mays*	Turtle Mountain White Corn	Turtle Mountain	Rare variety, needs to be reunited with home community	USDA North Central Regional Plant Introduction Station – Ames, IA	4
*Phaseolus vulgaris*	Hidatsa Red Bean	Three Affiliated Tribes—Hidatsa	Aligns with wish to honor the Dakota land we are growing on	Seed Savers Exchange – Decorah, IA	4
*Cucurbita pepo*	Algonquin Long Pie Pumpkin Squash	Known affiliation with Abenaki but likely connected to many tribes in the Northeastern US	Rare variety, needs to be reunited with growers, appealing taste and well-suited to 3SI	Sierra Seeds Cooperative – Nevada City, CA	2
*Helianthus annuus*	Arikara Sunflower	Three Affiliated Tribes—Arikara	Aligns with wish to honor the Dakota land we are growing on	USDA North Central Regional Plant Introduction Station – Ames, IA & Seed Savers Exchange – Decorah, IA	N/A^a^

^a^Not included in experimental design, but used as a border crop for the entire experiment per traditional Native grower practices

Our initial plans for 3SI methods and variety selection were two-pronged: we worked with our Advisory Board to identify Dakota varieties in honor of the history of the land on which ISU-3SI is located. Additionally, the varieties selected would need to be reunited with their home communities. We sent the seed index of Native varieties available through GRIN and to our Native collaborators. The maize variety “Turtle Mountain White” was identified as a Sister needing to be rematriated. We were able to obtain 1750 seeds of this accession from the North Central Plant Introduction Station (NCPIS) in Ames, IA. The remaining Sisters were chosen to complement this maize in the 3SI (Table 1).

Our original bean variety was the Hidatsa bean, housed at the USDA Western Regional Plant Introduction Station (WRPIS), in Washington State. However, we were not able to receive this accession in the quantity required for the full experiment. We were also not able to obtain the Arikara squash we had identified from the USDA from the NCPIS. While we were able to obtain seeds for our fourth sister, the Arikara Sunflower, we were only able to receive a portion of what we required. These obstacles led us to broaden our search to Seed Savers Exchange (SSE), of Decorah, IA, who have collaborated with Native Nations to support rematriation efforts using their extensive seed collection.

SSE generously donated their catalog Hidatsa Red bean variety to us. Though not the original seed we had selected, she was well-suited for the Three Sisters and was in need of rematriation. SSE also supplied us with the remaining quantity of Arikara sunflower. In our search for an Arikara squash however, we were directed to Rowen White, who sits on the board of SSE and is also a founding member of Sierra Seeds Cooperative, of Nevada City, CA. It is through her that we were introduced to the Algonquin Long Pie Pumpkin, a rare Abenaki heirloom in their collection. While this pumpkin may not be geographically similar to our other crops, she was well-suited to the goals and intentions of our project, and is rare and in need of being reunited with Native growers in the Northeast United States. Thus, it is through these relationships and collaboration with seed collection organizations across the Midwest US and further, that our project found the Three Sisters for the ISU-3SI.

As part of our collaborative work, our team has been in conversation with many Native seed-keepers, growers, and leaders involved in the rematriation movement. The process of rematriation is still evolving, and discussions on whom to appropriately identify as the initial recipient of these seeds within each community is ongoing. Given the nature of intertribal interaction through trade, intermarriage, and migration, ascribing each seed to a single Native nation is overly simplistic. At the same time, the process of collecting seeds usually involved assigning one tribal affiliation to each seed. Therefore, today the seed repositories we work with rarely identify more than one cultural group affiliated with each seed. Ideally, seed identification would happen through more in-depth historical research, including ethnographic work in communities. During a global pandemic, such efforts and logistics become even more complex. Not being able to visit communities for the homecoming of these seeds and visit with elders in person about their seed memories is a disappointing reality, but we are hopeful that the initial relationships created during this growing season will lend to future celebration and collaboration.

The Turtle Mountain White Corn is in the process of being reunited with her home community, the Turtle Mountain Band of Chippewa, located in Belcourt, ND. The Arikara sunflower and Hidatsa bean are also on their way home to the Three Affiliated Tribes, New Town, ND. The Algonquin pumpkin is being included in seed bundles distributed by the Indigenous Seed Keepers Network, a program within the Native American Food Sovereignty Alliance (Scandia, MN). Such relationship-building is a significant part of the collaborative research process, and as non-Native researchers, we have a responsibility to ensure that these seeds return home in the most culturally appropriate manner. We also have a responsibility to reciprocate for the valuable knowledge shared with the project. Rematriation is one way to fulfill that reciprocal obligation.

Another instructive example of the importance of our Advisory Board to the collaborative research process is our discussion of the possibility of nutrient analysis on samples of maize, beans and squash seeds. The board considered our ideas and request; however, we received mixed responses. Some members felt that we might receive valuable information through the process. After calling an advisory board meeting to discuss the implications of seed nutrient analysis, the Advisory Board decided that because seeds are considered cherished ancestors and relatives, submitting them for destructive nutritional analysis without the consent of culturally affiliated seed keepers would be inappropriate and disrespectful. Therefore, maize and beans were not analyzed for nutrient content given this consideration. Through compromise, we were granted permission to study the flesh, but not seeds, of squash. This conversation is on-going as we seek to bring in seed keepers from each community affiliated with the seeds, but collaborative research requires respect for all viewpoints. We seek consensus on such sensitive issues and doing so, and prioritizing this, only strengthens the collaborative research efforts.

### Ethnographic methods

We cleared the broader project and our methods with the ISU Internal Review Board to ensure protection of our participants. Our ethnographic methods used a critical and decolonizing approach to learn more about the meaning behind Indigenous agricultural practices (Bejarano 2019; Madison 2011; Ninomiya et al. 2020). This methodological technique of ethnography requires participant observation (Musante and DeWalt 2010). This involves spending time in a community, getting to know its members, and learning about cultural practices by watching and participating when asked. This methodology acquires data through careful observation and actively engaging with a community. The technique helps the researcher gain in-depth cultural knowledge because active engagement helps build trust between participants and researchers and provides first-hand experience. Prior to the beginning of the COVID-19 pandemic, team members traveled to Native communities and assisted with tilling, weeding fields, harvesting, soil testing, sharing meals, and participating in cultural celebrations.

To ensure that we could use critical ethnographic methodologies, Gish Hill has spent years developing relationships with Native growers in each of the participating communities. She initially reached out to Native growers in 2013 with a seed index of Indigenous seeds held at the PI station in Ames. Over time, Native growers affiliated with their nation’s food sovereignty programs began conversations with Gish Hill about the kinds of research needed in their communities. Gish Hill then approached the faculty at ISU to pull together a team that could address the needs Native growers shared with her. While the team is conducting on-going conversations with growers at Menominee nation in Wisconsin and Meskwaki nation in Iowa, we have conducted ethnographic work including interviews and participant observation in collaboration with NICC, Dream of Wild Health, and the Oneida nation of Wisconsin. We use the snowball sampling technique to recruit participants (Heckathorn 2011), allowing growers who are already part of the project to introduce us to new growers who might want to share their experiences in food sovereignty work.

Conducting formal interviews is another central component of ethnographic research. Interviews with Native growers and community members were conducted in 2019 and 2020. After informing a participant of the purpose of the research and reviewing how his or her knowledge will be used and protected, we gained consent to conduct and record an interview. We created a series of questions about growing techniques, the cultural importance of agriculture, seeds, soil, and food sovereignty. While interviewers used this question list during an interview, we encouraged the participant to share whatever they wish about a given topic and to take the interview in whatever direction they felt was relevant. In this way, we minimized the cultural bias that would affect the data if only the interviewer directed the conversation. While it is ideal to conduct interviews in-person, COVID-19 forced us to develop creative ways to complete our research. It was a struggle to foster the reciprocal relationships required to engage in ethical research with Native communities without being able to visit face-to-face. During isolation and social distancing, we continued to engage with communities by conducting interviews over video calls and staying up to date on community activities through social media. Recently, we have been able to return to the communities to continue face-to-face ethnography.

Once an interview was completed, we transcribed the recording word for word and returned the transcript to the interviewee. This provided each person with the opportunity to correct any misunderstandings, add to the transcript, or retract information they wish to remain private. Once the interviewee had the chance to review the transcript, we then proceeded to code each interview by hand, looking for common themes and terminology. Using coding software, e.g., NVivo, did not seem necessarily, useful, or appropriate in that respondents often used quite different language to describe similar ideas. In order to ensure that we did not misconstrue the meaning of what each participant shared in our analysis, we returned each draft article to our participants and the Advisory Board before submitting the manuscript.

### Community science as part of collaborative science

To fulfill the mission of collaborative Indigenous-centered research, we engaged Native growers in conducting their own 3SI research trials by monitoring and collecting their own data (Fig. 2). This connection between the ISU-3SI Research Experiment and the Native collaborators’ 3SI trials is similar in approach to the “mother–baby,” also called “hub-and-spoke,” experimental approach (Snapp et al. 2002, 2019). Here we integrate the “baby” trials with the “mother” through the Advisory Board, extension activities, and visits with the Native growers. This model can be thought of as a more integrated, intimate form of community science whereby community science is feeding back to inform the mother (or hub) experiment, and also allows for a closer connection between lead researchers and community members conducting experiments and collecting data. Many community science models use an app or website where the data typically moves in one direction—from community scientists to the data-synthesizers or lead researchers (usually university faculty). Here the data, synthesis, and collaboration work in all directions to benefit of all.

Evidence shows that engagement in community science increases the likelihood of the community scientists practicing conservation practices and has ripple effects through their social networks (Cooper et al. 2007; Ellwood et al. 2016). The use of community science to improve public understanding and conservation has been successfully used in ornithology (Hurlbert and Liang 2012), forest ecology (Mayer 2010), invasive plant ecology (Crall et al. 2013), and even water quality monitoring (Mullen and Allison 1999). However, such an approach has not been applied to soil health in peer-reviewed research. With growing interest in soil health (Roesch-McNally et al. 2018), and emergence of low-cost, yet scientifically robust methods to measure it, we engage Native growers in soil health community science. A community science network amongst ISU researchers and Native growers will not only have educational, cultural, and conservation benefits; but given time to generate enough data could also further our basic scientific understanding of intercropping effects on crops and soils.

Community scientists were recruited through networks already established through previous work by Gish Hill. We developed the experiment in conversation with our Advisory Board (many of whom were also community science participants). We distributed a document brief summarizing the mother-baby experiment and the role of community scientists in the broader project. To increase participation, we used a modified criteria. First, the participants had to grow each Sister independently and together in a minimum of 5 × 5 m plots. The replication number, varieties, and other management practices could vary. We requested that participants be consistent across the monocrop and intercropped plots. For example, if they irrigated then they irrigated all monocrops and the Three Sisters equally.

We worked with community scientists and the Advisory Board to determine what data was of greatest interest to all parties and which sampling methodologies would be possible and culturally appropriate. We asked collaborators to collect a soil samples at initiation of the experiment. These samples were sent to commercial soil test lab for analysis with results (and interpretation) provided to our community science collaborators. Community-scientist collected data included: plant height, soil moisture, aggregate stability, earthworm (*Lumbricus* spp.) abundance, and decomposition rate. Due to COVID-19, participation in the first year of the project was low and we did not collect enough community science data for analysis.

### Main site description and experimental design

The agronomic portion of this project, known as the ISU-3SI Research Experiment, was carried out at the Iowa State University Horticulture Research Station in Ames, Iowa (42.106778 N, 93.589583 W) on certified organic land. The soils are derived from Wisconsinan glacial till, and primarily as a Clarion loam soil series (fine-loamy, mixed, superactive, mesic Typic Hapludolls). Mean (± standard deviation) soil pH is 6.9 ± 0.2, and soil organic matter is 2.7 ± 0.4%. Fifty-year mean annual temperature for the area is 9.5 ± 1.0 °C, and annual precipitation is 895 ± 215 mm (IEM 2020).

**Fig. 3:**
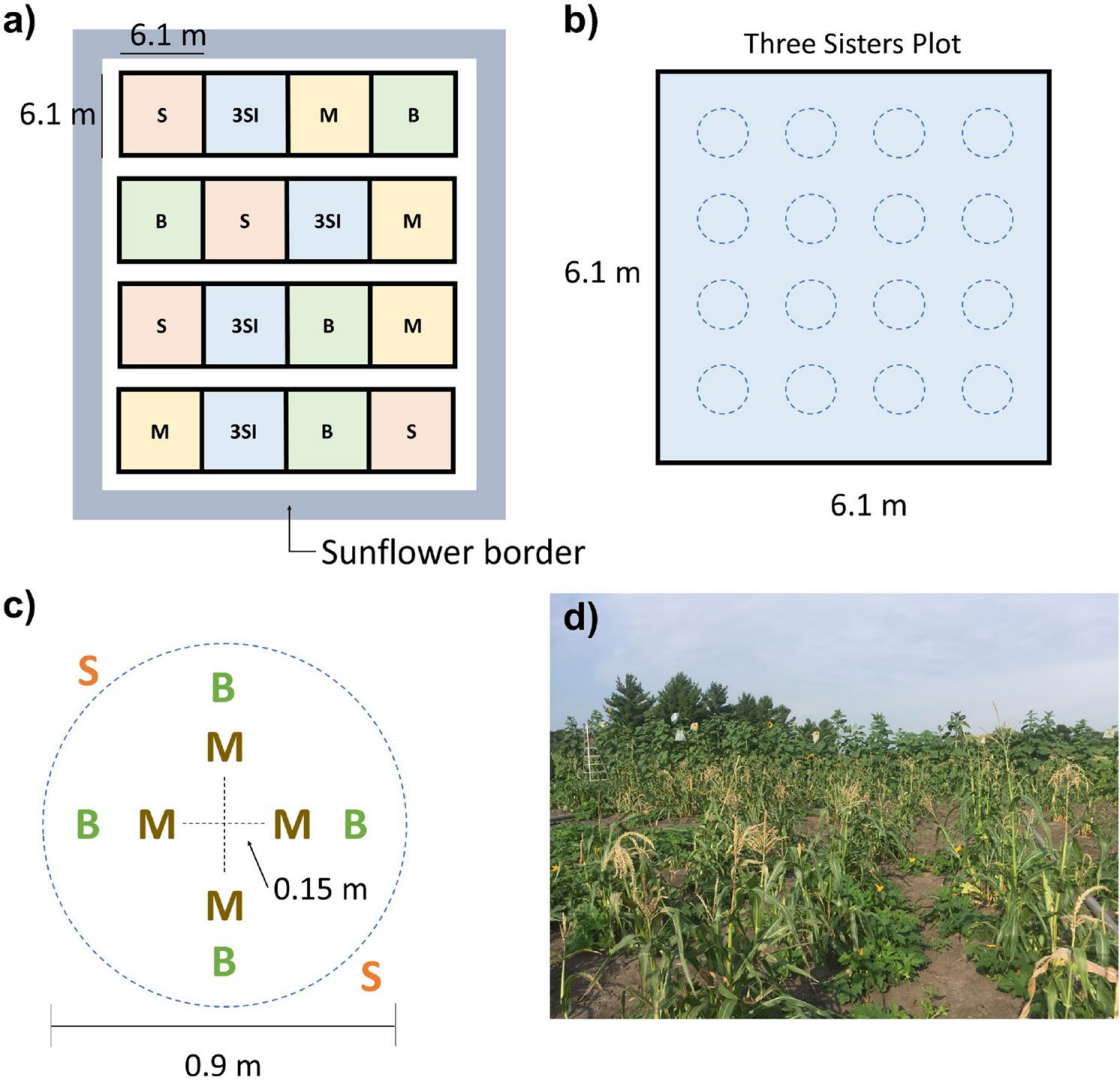
**a** The Iowa State University Three Sisters intercropping Research Experiment (ISU-3SI) showing monoculture maize, beans, squash, and Three Sisters intercropping (3SI) with sunflower border. **b** Dimension and layout of one 3SI plot with 16 mounds. **c** Dimension and layout of one mound within the 3SI plot. Monoculture mounds are the same layout without other crops. **d** Photo: E.M. Herrighty photo of ISU-3SI near Story City, IA

The treatments include three crops: monoculture maize (*Zea mays*), monoculture beans (*Phaseolus vulgaris*), monoculture squash (*Cucurbita pepo*), and a mixture of the three crops in 3SI. Four replicates of each of these four treatments were arranged in randomized complete block design (Fig. 3a). The border of the experiment was planted with sunflower (*Helianthus annuus*), which is considered a fourth sister in many communities (Mt. Pleasant and Burt 2010). Each treatment plot was 6.1 × 6.1 m and contained 16 mounds (0.9 m diameter and 0.2 m high) arranged in a grid (Fig. 3b). These mounds were created by furrowing soil and forming four mounds per furrow. The layout and planting of each mound, within plots, was based on Indigenous configurations of 3SI practice used in several communities throughout the Midwest (Wilson 1987; Kruse-Peeples 2016), and included four maize, four bean, and two squash plants (Fig. 3c). More information on cultivars, tribal affiliation, selection criteria or rationale, and seed source can be found in Table 1. Composted cattle manure was spread in the entire experiment, before creating mounds, at the rate of 22.4 ton ha^−1^. Each mound also received an organic slow-release fertilizer (Suståne^®^ 8-2-4, Cannon Falls, MN).

### Plant and soil collection and analyses

On August 28–30, 2020 maize was harvested early due to an extreme weather event (Derecho) and to salvage crops. No data could be collected on maize due to Derecho damage and severe smut infestation. Squash was harvested on October 12. Bean harvest occurred between October 15–20th depending on pod dryness. Bean and squash yields were collected from all 16 mounds from each replicated treatment. Beans and squash were stored in dry location for up to a week, weighed, counted, and numbers are represented for each plot. One squash was chosen randomly from each monoculture and 3SI replication. The seeds were removed for rematriation, and the inner flesh (excluding skin and seeds) was homogenized, and approximately 300 g for each sample were submitted to A&L Greatlakes Laboratories, Fort Wayne, Indiana, for tissue nutrient analysis. The analyses included crude protein, nitrogen, crude fiber, calcium, potassium, magnesium, phosphorus, sulfur, aluminum, boron, copper, iron, manganese, and zinc concentration. Certified standards were used for calibration of the instruments. After open vessel microwave digestion (SW846-3050B), samples were analyzed by Inductively Coupled Argon Plasma for minerals following AOAC 985.01 methods (AOAC International 2012a). Nitrogen and crude protein assessment was performed by the Dumas combustion method (AOAC 990.03) using an Elementar Rapid N Analyzer and LECO TRUMAC Carbon:Nitrogen Analyzer (AOAC International 2012b). Crude fiber was determined using method 32-10 (AACC, 10th edition, 2000) which consists of chemical digestion and subsequent combustion.

On August 28th, we collected homogenized soil samples comprised of cores (15 cm deep, 4.1 cm diameter) collected from the 16 mounds within individual plots. The fresh soils were sieved (< 2 mm) and analyzed for several soil chemical and biological properties. Soil nitrate was analyzed colorimetrically using the single-reagent method (Doane and Horwáth 2003). Soil test phosphorus (STP) and potassium (STK) were analyzed using Mehlich 3 extraction (Mehlich 1984), soil test sulfur (STS) was extracted using phosphate extraction, and all extracts run on a ion coupled plasma optical emission spectrometer (5800 ICP-OES; Agilent, Santa Clara, CA). Soil CO_2_ burst was analyzed using the updated Solvita^®^ paddle and reader method (Haney and Haney 2010; Yost et al. 2018). Microbial biomass carbon (MBC), microbial biomass N (MBN) and salt-extractable C (SEOC) were measured using a 0.5 M K_2_SO_4_ extraction with the chloroform fumigation-extraction method (Vance et al. 1987; Brookes et al. 1985); and corrected using 0.45 and 0.54 extraction efficiency (Brookes et al. 1985; Joergensen 1996). MBC, MBN, and SEOC extracts were then analyzed for non-purgeable organic C and total N by catalytic oxidation combustion (Shimadzu TOC-L analyzer, Shimadzu Corporation, Columbia, MD, USA).

### Data management and analyses

All project members were trained and abide by ISU Internal Review Board oversight on ethical and privacy concerns related to knowledge shared by research participants. Interviews were recorded with digital recorder, transcribed, and coded by hand. All agronomic data were analyzed in R v.3.4.3. (R Core Team 2018). Data were checked for normality and homogeneity of variances, and all data conformed to standards to proceed with parametric analyses. We used simple one-way analysis of variance using the *aov* function in R to determine differences among monocrops and 3SI. Our focus was comparing average of the three monocrops with the 3SI treatment, thus we used orthogonal contrasts to make this comparison. Significant treatment effects are determined at α = 0.1, 0.01, 0.001 for marginal significance, significance, and highly significant respectively. Means of data are presented with standard deviations or with replicate values to express variance. Data were visualized in SigmaPlot v.14 (Systat Software, Inc.; San Jose, CA).

#### Results and discussion

### Cultural importance of Three Sisters and sustainable agriculture

In our ethnographic work, Native growers have underscored the importance of rejuvenating the Indigenous agricultural practices of their nations as a path towards food sovereignty. Considering that food deserts are a reality for many Native nations (Pindus and Hafford 2019; Warne and Wescott 2019), regaining access to culturally appropriate food is integral to a healthy and sustainable food system (Mihesuah and Hoover 2019). Food sovereignty ensures the right of communities to shape food policy. More specifically, it posits that we all deserve to be able to eat healthy, nutritious, and culturally valued foods and to be able to acquire them in culturally appropriate ways (Patel 2009; Desmarais and Wittman 2014; Gish Hill 2017). An Omaha grower who has worked with the Omaha nation schools and the Center for Rural Affairs, SF, states, “Food sovereignty, to me, is the right of the people to have their own food choices, to be able to define their food system.” RW, an Oneida of Wisconsin grower who has started her own farmstead and is a founding member of both Ohelaku Corn Growers Co-op, and a new bean growing co-op, states, “it [food sovereignty] means that our people have access to culturally appropriate foods and a place to grow them. We can harvest consume, prepare and preserve our foods in a way that is culturally appropriate to our people. All the way from seed saving, to planting, to preserving the food.” We have found that in Native communities throughout the Midwest, working towards food sovereignty involves sharing knowledge between generations, especially about the return seeds to their home communities (or rematriation), and the repair of the relationship between people and the broader ecosystem, particularly soil and water.

One result of rejuvenating Native agriculture practices, like growing Three Sisters within these communities, is that the gardens provide a space for intergenerational transfer of knowledge. RW is part of the Ohelaku Corn Growers Co-op, a multigeneration group that brings together elders and children, even very young ones, to pass on agricultural TEK. She shares that sometimes four generations of one family are in the barn husking corn together. RW continues, “when we do have the youth working in there, we joke that it’s not child labor, it’s passing on traditions.” SF notes that she has been amazed by how many elders want to grow and in the process, “remember and tell their stories about when they were kids, the harvest celebration when all the people would bring in their harvest and share it with the people.” Working together on weeding or harvesting has become a powerful way to pass on cultural, historical, and agricultural knowledge from elders to children.

Rejuvenating Native agriculture requires Indigenous seeds, and as a result, there has been a rapid growth of the seed rematriation movement. Several growers have shared with us the importance of this movement and discussed their role in it. The evolving concept of rematriation in Native North America is an expansion and reframing of the repatriation work defined by the Native American Graves Protection and Repatriation Act (NAGPRA) of 1990 (Gish Hill 2017). The rematriation movement, as defined by Rowen White, a Mohawk seedkeeper, more appropriately recognizes the gendered nature of gardening in many Native cultures (White 2018). Rematriation acknowledges the land, Mother Earth, as a central figure within Indigenous agricultural systems. Additionally, the term recognizes the role of women as gardeners and seedkeepers in most Native agricultural communities as well as seeds as feminine entities themselves. While very rarely, a Native nation may view some plants such as corn as male, almost all nations entrust seeds to women, including Hopi where men farm (Nabhan 2002). We use the term “rematriation” because this is the term our Native collaborators prefer. In returning valued seeds to Native growers, communities are able to revitalize and reclaim their cultural food ways. Seeds, cherished relatives and ancestors to Native growers, are crucial members of these systems and traditions (Gish Hill 2017). Healthy, culturally appropriate, and sustainable futures of Native growers require returning ancestral seeds.

Often the Native growers we collaborate with express the importance of Native agriculture as a method to repair the relationships humans have with the broader ecosystem. In their traditional cultural teachings, the Nations collaborating in this project view Earth as a caregiver, and soil specifically as the nurturing entity from which all life is born. These growers assert the importance of protecting the health of the global ecosystem (including abiotic and human components). They express that sustainable land use is a high priority undergirded by embedded cultural concepts tying land use practices to the impacts on health of future generations. JG, a Ho-Chunk grower and manager of Dream of Wild Health in Minnesota, states, “Soil to me is just an alias for the earth.” She continues that from an Indigenous perspective, the earth is our Mother. Fertile top soil, where seeds are placed, is seen as her womb. She explains, “It’s knowing that we need to have a good healthy place for our seeds to go. Overtime she’s [Mother Earth] been depleted and different chemicals poured onto her and just not being respected in a good way.” Many growers shared with us the importance of caring for the land and soil. JG explained this as, “wanting to be able to help the soil heal and regenerate it—get some of those good micro and macro biology back into the soil to help our entire ecosystem, and give our seeds a good place to live.” Our Native collaborators understand plants, seeds and soil to have animacy. Kimmerer (2015) notes that in many Native languages the barriers between human and non-human (such as plants, animals, and landscapes) are dissolved through use of pronouns for all of nature, indicating animacy for all these entities. While English does not use pronouns for the rest of nature, using “he” or “she” to refer to the Earth, plants, and seeds is a way to reference this perspective. Our research seeks to foster a wider understanding of this perspective of plant, soil, and human relationships.

For the Native growers we are working with, rejuvenating their traditional agricultural practices has had a profound impact on the well-being of their wider communities. For SF, she sees this impact in, “reclaiming our identity and in asserting who we are.” She also noted that rejuvenating these practices helps her to “reclaim my identity and what had been taken away from previous generations, like my grandparents generations.” JG states, “The blood, sweat and tears of what I put into my field I know is also going to help my people in the long run. And it’s going to help make stronger seeds too.” SF notes, “I can go out and feed myself out of the timber and the prairie by the river,” but she notes that not everyone in her Native Nation is able to do that. She grows to make Indigenous maize and other foods available to everyone in her community. JG shares, “why I garden is for life.” She had realized that non-Indigenous foods are killing her people (e.g., heart disease, diabetes, cancers, etc.) and says she wanted to be able to help fix that. All these growers are working to make the healthy, nutritious Indigenous foods they grow a staple for their people.

### The effect of Three Sisters intercropping on plants and soils after 1 year

The first year of the ISU-3SI Research Experiment was challenging. The site received a total annual precipitation of 683 mm (compared to MAP of 895 mm), and 61% of this occurred during the growing season. On August 10, 2020 a Derecho, a hurricane-like inland storm, hit central Iowa and many other states (Hosseini et al. 2020; Halverson 2021). This Derecho created surface wind speeds greater than 44 m s^−1^ at a nearby weather station. These high-speed winds lodged maize throughout Iowa, including the maize grown in the first year of the experiment. The Derecho hastened harvest, so both plant and soil collection occurred earlier than expected. In the case of maize, the crop was completely damaged, but we were able to salvage some seeds for rematriation.

Intercropping the Three Sisters lowered both crop weight and marketable numbers compared to monoculture (Table 2). This was expected since there is greater plant competition for solar radiation, water, and nutrients in the 3SI than monoculture treatment; and several studies have shown a net decrease in yield with intercropping (Wolff and Coltman 1990; Wu et al. 2016). However, the benefits to intercropping are better measured with the land equivalent ratio (LER, Mead and Willey 1980). This metric accounts for sum of all crop yields based on equivalent area, and LER values greater than 1 suggest that the combined intercropped yield is saving that fraction of additional land for the same amount of grain production with monoculture cropping.

**Table 2:** Crop yield as marketable number and weight

Crop	Marketable number		3SI % of mono. Total	Marketable weight (kg)		3SI % of mono. Total
	Monoculture	Three Sisters intercropping (3SI)		Monoculture	Three Sisters intercropping (3SI)	
Maize^a^	ND	ND	ND	ND	ND	ND
Bean	2569 ± 374a	225 ± 49b	8.5 ± 2.4	0.71 ± 0.10a	0.06 ± 0.01b	9.0 ± 2.9
Squash	96 ± 10a	70 ± 5b	73.3 ± 4.9	192.3 ± 24a	133.3 ± 14.8b	69.4 ± 3.1

Mean ± standard deviation (n = 4), comparisons within rows and lower-case letters show significance at *p* value ≤ 0.1

^a^No data (ND) available due to Corn Smut (*Ustilago maydis*) and Iowa Derecho damage (Hosseini et al. 2020)

Despite the Derecho damaging all maize yield, the overall productivity of 3SI was greater than monocrops based on LER. We estimated the 3SI maize yield after the Derecho to be 50–75% of the monocrop. By using these maize estimates our total LER would have been between 1.28 and 1.53 ± 0.03. Three previous intercropping meta-analyses covering 90 to 100 publications, many of which are overlapping, showed that mean LER ranges from 1.22 to 1.32 (Yu et al. 2015; Martin-Guay et al. 2018; Xu et al. 2020). Thus, our LER meets or even exceeds the average of most previous intercropping studies. Raseduzzaman and Jensen (2017) also demonstrated that intercropping increased yield stability, or in other words, decreased the temporal variability in yield driven by climate, pest, and other environmental factors. Yield stability is becoming increasingly important to growers as climate change increases extreme weather events in the Midwest US and elsewhere (Thomson et al. 2014; Gaudin et al. 2015; Liu and Basso 2020). We hope to monitor differences in yield stability amongst 3SI and monocrops.

3SI did not affect nutrient content in the edible squash tissue compared to monocropped squash (Table 3). Thus, we would provisionally reject our hypothesis that intercropping would increase nutrient density. However, due to small sample size and the environmental challenges of this first growing season, these results should be interpreted with caution. Some studies of intercropping have focused on yield and nutritional adequacy when 3SI is used in milpa systems of Mesoamerica (DeYoung et al. 2017; Lopez-Ridaura et al. 2021). For example, a recent study surprisingly showed no yield penalty for intercropping maize in Western Highlands of Guatemala across 357 plots, but did show an increase in caloric density of the maize and potential nutritional value for the intercropped systems compared to monoculture maize (Lopez-Ridaura et al. 2021). Thus, enhanced nutrient density and even comparable yields are possible in regions where Three Sisters has remained a vital source of food (Hellin et al. 2017; Lopez-Ridaura et al. 2019). Nutritional density across crops, but also within a crop, will continue to be an important area of exploratory research and critical ecosystem function of intercropping through providing nutrient density and diversity.

**Table 3:** Mean ± standard deviation of nutrient concentrations in flesh of pumpkin squash (Algonquin Long Pie Pumpkin) planted in monoculture or Three Sisters intercropping

Nutrient in squash flesh	Monoculture	Three Sisters intercropping
Nitrogen (%)	1.56 ± 0.06	1.55 ± 0.24
Crude protein (%)	9.55 ± 0.63	9.65 ± 1.49
Crude fiber (%)	17.88 ± 0.74	18.48 ± 2.49
Calcium (%)	0.18 ± 0.02	0.19 ± 0.03
Potassium (%)	3.95 ± 0.58	4.04 ± 1.39
Magnesium (%)	0.11 ± 0.01	0.09 ± 0.01
Phosphorus (%)	0.30 ± 0.03	0.28 ± 0.07
Sulfur (%)	0.18 ± 0.02	0.19 ± 0.03
Aluminum (ppm)	5.5 ± 4.4	2.8 ± 1.0
Boron (ppm)	33 ± 2.94	33 ± 4.08
Copper (ppm)	4.3 ± 0.5	4.8 ± 1.5
Iron (ppm)	26.3 ± 6.1	25.8 ± 7.4
Manganese (ppm)	5.8 ± 1.5	4.8 ± 1.0
Zinc (ppm)	11.8 ± 1.0	10.0 ± 2.5

**Fig. 4:**
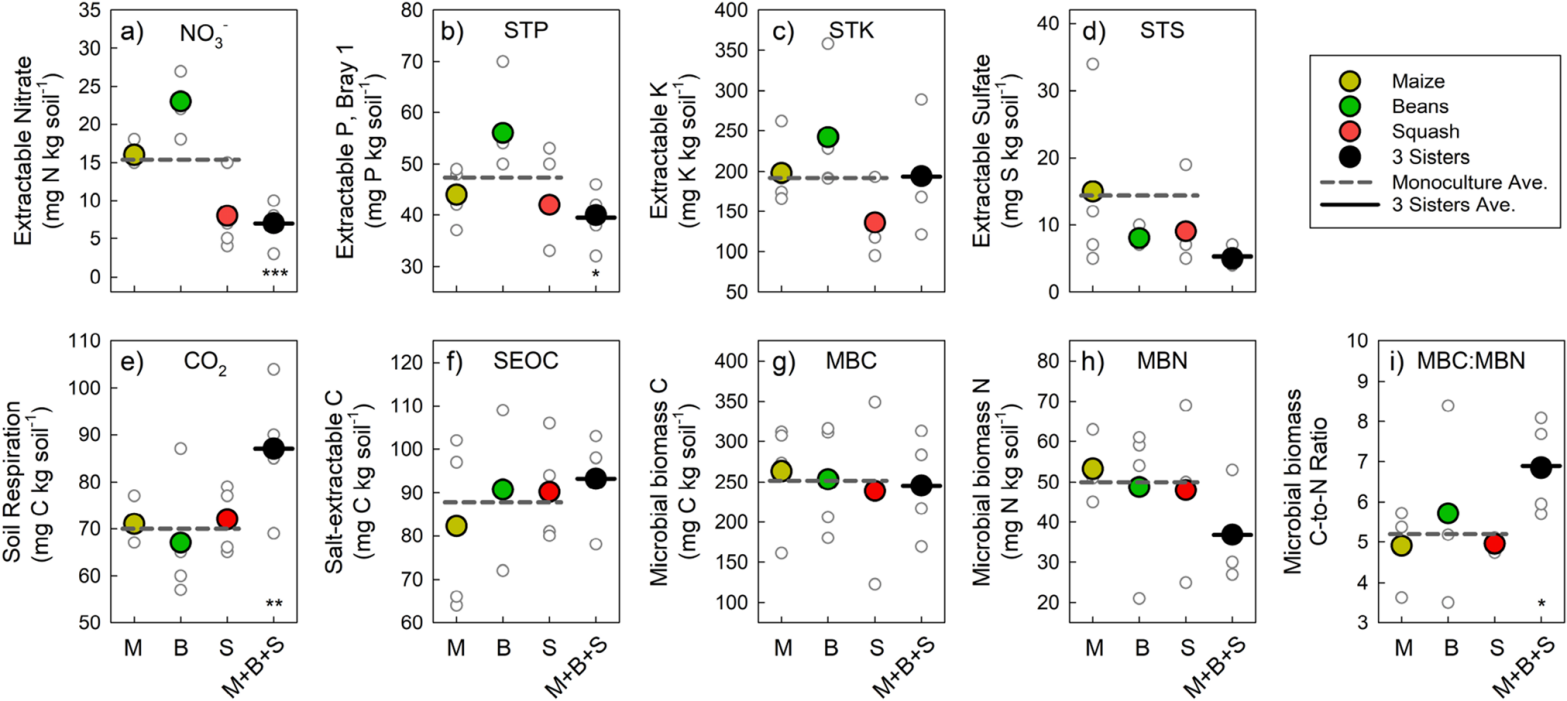
Soil properties measured on 15 August 2020 at the Iowa State University Three Sisters intercropping Research Experiment (ISU-3SI). **a** Soil extractable nitrate, **b** soil test phosphorus with Bray P1 extraction, **c** soil test potassium with Mehlich III extraction, **d** soil test sulfur with phosphate extraction, **e** 24 h soil respiration with air-dried, rewet soils, **f** salt-extractable organic C, **g** microbial biomass C extracted with chloroform-fumigation extraction, **h** microbial biomass N extracted with chloroform-fumigation extraction, **i** microbial biomass C-to-N ratio. Replicates for each treatment shown with open circles (n = 4), and significant differences between monoculture vs. Three Sisters intercropping (M + B + S) indicated by asterisks (*< 0.1, **<0.01, ***<0.001)

The 3SI had some significant effects on soil properties after just 1 year (Table 4; Fig. 4). We hypothesized that intercropping would decrease nutrient concentrations at the end of the growing season, especially those nutrients most mobile in soils (e.g. nitrate and sulfate), compared to average of monocrops. 3SI decreased extractable soil nitrate by 54% compared to monocrop treatments, supporting our hypothesis. Although not statistically significant, 64% lower extractable sulfate adds additional support to our hypothesis since sulfate is also a mobile, plant macronutrient. Concentrations of less mobile plant-available macronutrients, measured with STP and STK, were no different between monocrop and intercropping. Since the soils had similar concentrations of plant-available nutrients to begin the growing season, lower concentrations at harvest suggest a more efficient use of these nutrients by plants, especially with those nutrients that are easily lost via leaching or greenhouse gases (e.g., nitrate-N and sulfate-S). Because nitrate-N is a major contributor to local and regional water quality issues (Turner and Rabalais 2003), this suggests that 3SI may lessen the impact on water quality compared to monoculture cropping.

**Table 4:** Summary table of soil properties under monoculture and Three Sister Intercropping with description, means, treatment effect and significance

Soil variable	Description and importance	Variable units	Monoculture mean	Three Sisters intercropping mean	Δ 3SI (% change from intercropping)	Significance (p value)
Static soil properties						
Organic matter	Concentration of organic material measured with loss on ignition	%	2.7	2.8	+ 1	ns
pH		Unitless	6.83	6.89	+ 3.1	ns
Bulk density	Mass of soil per volume	g cm^−1^	1.23	1.22	− 1.4	ns
Dynamic chemical soil properties						
Gravimetric water content	Water as fraction of dry soil	g H_2_O g dry soil^−1^	0.179	0.184	+ 2.8	ns
Nitrate-N (NO_3_^−^)^a^	Plant-available N, also highly mobile in soils	mg kg dry soil^−1^	12.4	15.4	− 54.7	< 0.001
Soil test phosphorus (STP)^a^	Plant-available form of P	mg kg dry soil^−1^	47.4	39.5	−16.9	0.067
Soil test potassium (STK)^a^	Plant-available form of K	mg kg dry soil^−1^	192	194	+ 0.9	ns
Soil test sulfur (STS)^a^	Plant-available form of S	mg kg dry soil^−1^	14	5	− 63.6	ns
Biological soil properties						
CO_2_ burst (CO_2_)^a^	CO_2_ released from an air-dried soil when rewetted	mg kg dry soil^−1^	70	87	+ 24.3	0.019
Salt-extractable organic carbon (SEOC)^a^	Organic C extracted with 0.5 M K_2_SO_4_. Represents low-molecular weight C compounds and usually healthier soils have more SEOC	mg kg dry soil^−1^	88	93	+ 0.1	ns
Microbial biomass carbon (MBC)^a^	The concentration of microbial biomass C, or C in living organisms not seen with naked eye	mg kg dry soil^−1^	252	246	0	ns
Microbial biomass nitrogen (MBN)^a^	The concentration of microbial biomass N, or N in living organisms not seen with naked eye	mg kg dry soil^−1^	43	36	− 0.1	ns
Microbial biomass carbon-to-nitrogen ratio (MBC:MBN)^a^	Ratio of microbial biomass C to N. Indicates potential C versus N supply/demand, but also community composition	unitless	8.32	6.85	− 0.2	ns

^a^Variables with individual plot values shown in Fig. 4

*ns* not significant at α = 0.1

Our findings align with a study conducted on Ultisol soils in Pennsylvania, US which showed 3SI increased nitrogen and phosphorus uptake in polyculture compared to monocrops, more or less regardless of fertilizer rates added (Zhang et al. 2014). Enhanced plant uptake of soil nutrients in 3SI is thought to be due to greater root foraging and differential niche partitioning due to diversity and complementarity of rooting architecture, morphology, and physiologies of each of the Three Sisters (Zhang et al. 2014). Furthermore, surprising interactions can emerge, where 3SI increased lateral root branching of all crops when grown together compared to when grown separately (Zhang et al. 2014).

Crop diversity, at least with regard to rotations (or diversity through time), has well-known strong effects on soil microbiota. Meta-analyses report average increases from crop rotational diversity on soil microbial biomass C by 21%, microbial biomass N by 26%, richness by 15%, and diversity by 3% compared to monoculture crops (McDaniel et al. 2014; Venter et al. 2016). The effects of intercropping diversity on soil microbial activity and biomass are less known, but a recent meta-analysis showed intercropping increased soil extracellular enzyme activities, catalysts produced by microorganisms and plants to acquire carbon and nutrients, by 13% on average (Curtright and Tiemann 2021). We hypothesized that 3SI would increase soil microbial biomass and activity measured as CO_2_ Burst. While intercropping increased CO_2_ Burst by 24% compared to monocrops, supporting our hypothesis, there was no effect of 3SI on salt-extractable organic C nor microbial biomass (Fig. 4). Greater microbial biomass and activity may reflect a more efficient use of new carbon inputs (like crop residue or rhizodeposits) and therefore indicate 3SI could perhaps have greater ability to sequester carbon over longer timeframes (Geyer et al. 2020). Furthermore, mounting evidence has shown that the microbial activity measured as CO_2_ Burst can be linked to maize N needs and thus potentially used to guide N fertilizer rates (Franzluebbers 2018; Yost et al. 2018).

The microbial biomass C-to-N ratio, thought to reflect N cycling dynamics and even microbial community composition (Strickland and Rousk 2010; Li et al. 2019, 2020, 2021), was 32% greater in 3SI compared to monocrops. The greater MBC:MBN in 3SI compared to monocrops could either be due to greater N immobilization by microbial biomass, greater abundance of fungi relative to bacteria, or both (Aoyama and Nozawa 1993; Strickland and Rousk 2010; Li et al. 2021). Links between microbial activity and plant nutrient uptake are further evidenced by a study showing increased importance of arbuscular mycorrhizal fungi (AMF) when plant roots intermingle between species, and the AMF become more important for plant nutrient uptake (Xiao et al. 2010; Qiao et al. 2016). A second year of above and belowground crop and soil dynamics will be measured in Year 2 of the ISU-3SI Research Experiment.

One aspect to consider here when comparing intercropping with monocropping is the labor involved. More labor is involved with intercropping due to growing multiple crops in close proximity (Ransom 1990; Gebru 2015; Dahlin and Rusinamhodzi 2019). This works for small-scale gardening or farming, like many of our collaborators’ operations, but it is more challenging when used at larger scales requiring mechanization. Intercropping creates complex canopy structures and makes mechanized harvesting very difficult. Thus, close-knit intercropping often requires precise weed control, hand-harvesting, and thus is currently largely limited to smaller scales. However, this does not necessarily mean that large-scale intercropping has to require high labor demands into the future. With improvements in image-recognition software and robotics used in agriculture for automated management and harvesting (Tian et al. 2020), it is imaginable that intercropping can be carried out at a large-scale with similar labor demands as monocultures.

## Conclusions

Collaborative multi-disciplinary research agendas, combined with community science surrounding the 3SI, in Native American communities has many benefits and some challenges. The goals and benefits include: (i) engaging underrepresented communities in enhancing and expanding their agricultural skills, (ii) rematriating seeds, and (iii) enhancing our basic understanding of agroecology of intercropping and potentially informing industrial agricultural practices. The challenges or limitations, however, include: (i) compromising with Native growers on what natural scientists considered routine scientific analyses due to important cultural considerations (e.g., not analyzing seeds for nutrient content); (ii) navigating the cultural differences among researchers in a multi-disciplinary team over things such as research methodologies, language, and expectations; and (iii) difficulty recruiting citizen scientists during a pandemic (e.g., COVID-19). The benefits of a project like this far outweigh any challenges, and the challenges in and of themselves are actually opportunities for learning and growth.

In the first manuscript from this project, we show that Native growers are concerned about food sovereignty, seed rematriation, and environmental issues. There are cultural institutions in place in Native communities for passing on TEK. However, rejuvenating Native agricultural practices like the Three Sisters as a component of the fight for Native food sovereignty is a relatively recent effort. Native growers involved in this work are fighting hundreds of years of agricultural loss and forced assimilation, and therefore can use support from researchers prepared to center Native research methodologies and reciprocity. A collaborative research project using 3SI shows potential for enhancing our understanding of intercropping, rejuvenating Native agricultural practices, and improving Native communities’ food sovereignty.

## Abbreviations

3SI: Three Sisters intercropping
LER: Land equivalent ratio
SDG: Sustainable development goal
SSE: Seed Savers Exchange
STK: Soil test potassium
STP: Soil test phosphorus
TEK: Traditional ecological knowledge
